# Sleep disorders related to index and comorbid mental disorders and psychotropic drugs

**DOI:** 10.1186/s12991-023-00452-3

**Published:** 2023-05-27

**Authors:** Ray M. Merrill, McKay K. Ashton, Emily Angell

**Affiliations:** Department of Public Health, College of Life Sciences, Brigham Young University, Provo, UT 84602 USA

**Keywords:** Claims data, Comorbid, Insomnia, Mental disorders, Psychotropic drugs, Sleep apnea

## Abstract

**Purpose:**

Mental disorders positively associate with sleep disorders. This study will explore the moderating influence of comorbid mental disorders and whether selected psychotropic drugs correlate with sleep disorders after adjusting for mental disorders.

**Methods:**

A retrospective cohort study design was employed using medical claim data from the Deseret Mutual Benefit Administrators (DMBA). Mental disorders, psychotropic drug use, and demographic data were extracted from claim files for ages 18–64, years 2016–2020.

**Results:**

Approximately 11.7% filed one or more claims for a sleep disorder [insomnia (2.2%) and sleep apnea (9.7%)]. Rates for selected mental disorders ranged from 0.09% for schizophrenia to 8.4% for anxiety. The rate of insomnia is greater in those with bipolar disorder or schizophrenia than in other mental disorders. The rate of sleep apnea is greater in those with bipolar disorder and depression. There is a significantly positive association between mental disorders and insomnia and sleep apnea, more so for insomnia, especially if they had other comorbid mental disorders. Psychotropic drugs other than CNS stimulants, primarily sedatives (non-barbiturate) and psychostimulants, explain much of the positive association between anxiety, depression, and bipolar disorder with insomnia. Psychotropic drugs with the largest effect on sleep disorders are sedatives (non-barbiturate) and psychostimulants for insomnia and psychostimulants and anticonvulsants for sleep apnea.

**Conclusion:**

Mental disorders positively correlate with insomnia and sleep apnea. The positive association is greater when multiple mental illness exists. Bipolar disorder and schizophrenia are most strongly associated with insomnia, and bipolar disorder and depression are most strongly associated with sleep disorders. Psychotropic drugs other than CNS stimulants, primarily sedatives (non-barbiturate) and psychostimulants for treating anxiety, depression, or bipolar disorder are associated with higher levels of insomnia and sleep apnea.

## Introduction

Sleep is a basic need that is essential to physical and mental health [1, 2]. Sleep disorders involve sleep disturbances related to inappropriate sleep, severe sleep deprivation, and pauses in breathing while sleeping. Sleep disturbances can result in daytime irritability, a loss of focus, memory, and other cognitive abilities, as well as a weakened immune system and increased rates of chronic illness [2–8].

Mental disorders associated with sleep disorders include stress, anxiety, depression, attention-deficit hyperactivity disorder (ADHD), bipolar disorder, and others [9–13], with the causal association often two ways [14, 15]. Sleep disturbances, especially insomnia, are common in people with schizophrenia or bipolar disorder [16]. One study found that 50% of the patients with schizophrenia also exhibited insomnia [17]; other research showed that slow sleep waves and altered sleep spindles are associated with clinical symptoms and cognitive impairments often reported in schizophrenia patients [18]. ADHD is frequently associated with circadian rhythm sleep disorders [19]. Delayed circadian rhythms have also been associated with OCD [20]. Depression is not only known to be a risk factor for insomnia, but insomnia is also seen as a risk factor for depression [21]. Arousal and alertness are heightened in people who are stressed and have anxiety [22]. All of these disturbances can affect sleep.

The positive association between sleep disorders and mental disorders observed in previous studies may be stronger if comorbid mental disorders are involved versus a single mental disorder. Comorbid mental disorders exist if two or more mental disorders occur in the same individual [23]. Cross-sectional survey results have found comorbid mental disorders to range from 45 to 54% [24–26]. Rates of comorbid mental disorders may be influenced by changes in diagnoses following the onset of the index disorder, as well as be due to shared characteristics underlying given types of mental disorders [27]. A population-based cohort study in Denmark showed an increased risk of developing other mental illnesses following an index mental disorder, growing stronger over time [27]. More severe mental disorders have also been associated with more comorbid mental disorders [28]. Whether a mental disorder with comorbid mental illness has a stronger association with insomnia and sleep apnea than a mental disorder without comorbid mental illness deserves further study.

Psychotropic drug use has also been associated with sleep disorders. Some antidepressants can negatively affect sleep, such as impairments of sleep, sleep apnea, and rapid eye movement (REM) sleep behavior disorder [29, 30]. Antidepressants can cause insomnia [29, 31]. Several antidepressants can suppress REM sleep [32]. While many antidepressants can have a negative impact on sleep, some antidepressants may improve sleep quality [33], and one antidepressant has been approved for treating insomnia [30]. However, these are exceptions among antidepressants. Some anxiety medications, including short-acting benzodiazepines, have been associated with lower sleep quality [34], but other benzodiazepines are used to treat insomnia [30]. Finally, studies assessing the direction of association between psychotropic medications and sleep disorders have been mixed [29, 35, 36].

The purpose of the current study is to identify the rate of insomnia and sleep apnea associated with selected index mental disorders and, for each index mental disorder, whether comorbid mental illness modifies the rate of insomnia and sleep apnea.

Mental disorders evaluated in this study include schizophrenia, bipolar disorder, depression, anxiety, obsessive–compulsive disorder, stress, and attention-deficit hyperactive disorder. In addition, we identify the level and type of medication used across the index mental disorders. We also consider whether higher levels of insomnia and sleep apnea are associated with psychotropic drug use after adjusting for selected demographic variables and mental disorders.

The current study is based on medical claims data. Because only those with more serious mental disorders and sleep disorders tend to seek treatment, rates of these disorders will be lower than found in cross-sectional surveys. Consider that in the United States, chronic insomnia affects 10–33% of adults, and obstructive sleep apnea affects 9–21% of women and 19–28% of men [37]. However, individuals with insomnia often do not seek medical treatment. A study in Spain found that only 12.2% of individuals with insomnia had an insomnia diagnosis registered in their medical records [38]. A report involving U.S. adults found that only about 25% of those with symptoms of obstructive sleep apnea pursue medical care [39].

Study hypotheses are:

1. Sleep disorders are positively associated with the selected index mental disorders stated above.
2. If a sleep disorder is positively associated with a mental disorder, the association is greater if the person has multiple mental disorders versus a single mental disorder.
3. Psychotropic drug use increases the prevalence of sleep disorders, after adjusting for demographic variables and mental disorders.

## Methods

### Study population

The current study involves contract holders in a large insurance database called the Deseret Mutual Benefit Administrators (DMBA). The company began in 1970 to provide health insurance and retirement income to employees and their families. Electronic claims data were available in the years 2016 through 2020. Contract holders reside in Utah (73%), Idaho (10%), other mountain states (4%), pacific states (8%), central states (4%), and eastern states (1%).

Each year the entire dataset consists of approximately 27% contract holders, 21% spouses, 48% dependent children, and 4% other (e.g., married child, stepchild, disabled dependent). Contract holders work in the Church education system, seminaries, and institutes (34%); as manual laborers (31%); in other companies (10%); and in other capacities (19%). Employee retention from year to year was approximately 92%. Insured individuals tend to be dropped from DMBA at ages 65 years or older as they retire from work and become eligible for Medicare. The linked database used in the current study was de-identified according to Health Insurance Portability and Accountability Act (HIPAA) guidelines.

### Data

The study involved DMBA contract holders aged 18–64 in 2016 (*n* = 20,655), 2017 (*n* = 21,360), 2018 (*n* = 21,835), 2019 (*n* = 21,352), and 2020 (*n* = 21,027). These data represent eligibility data linked to automated medical claims records using a common identifying number. The database was de-identified according to Health Insurance Portability and Accountability Act (HIPAA) guidelines. Ethical approval and informed consent to participate was waived by the authors’ institutional review board because the data were anonymized before assessment (IRB2021-157).

The International Classification of Diseases, Tenth Revision, Clinical Modification (ICD-10-CM) codes were used to classify mental disorders and sleep disorders [40]. Psychotropic drugs were classified as CNS stimulants, psychostimulants (antidepressants), amphetamine preparations, sedatives (non-barbiturate), ataractics (tranquilizers), and anticonvulsants.

Specific mental disorders (and their ICD-10-CM codes) included schizophrenia, delusional, and other non-mood-psychotic disorders (F20–F29) (hereafter, called schizophrenia); bipolar disorder (F31); depression (F32, F33); anxiety (F40, F41); obsessive–compulsive disorder (OCD) (F42); stress (F43); and attention-deficit hyperactivity disorder (ADHD) (F90). Sleep disorders were classified as any (insomnia, hypersomnia, circadian rhythm sleep disorders, sleep apnea, narcolepsy and cataplexy, parasomnia, and sleep-related movement disorders [G47]), as well as two common types of sleep disorders, insomnia (G47.0) and sleep apnea (G47.3).

Rates of specific types of mental disorders and sleep disorders consisted of the number of contract holders filing one or more claims for each of the conditions divided by the number of contract holders. If multiple claims were filed in a given year for a specific condition, it was only counted once in the numerator of the rate calculation. However, an individual could contribute to more than one type of condition in a given year.

Other variables considered in this study were age, sex, marital status, dependent children status, salary, and year. Classifications for these variables appear in Table 1.

**Table 1:** Odds of having a sleep disorder by demographic variables, 2016–2020

	No.	%	Any sleep disorder			Insomnia			Sleep apnea		
			Odds ratio^†^	95% LCL^†^	95% UCL^†^	Odds ratio^†^	95% LCL^†^	95% UCL^†^	Odds ratio^†^	95% LCL^†^	95% UCL^†^
Age											
18–29	12177	11.46	1.00			1.00			1.00		
30–39	23445	22.07	2.33	2.04	2.66	2.04	1.57	2.66	2.55	2.18	2.99
40–49	26415	24.87	4.58	4.03	5.21	3.69	2.87	4.75	5.27	4.53	6.14
50–59	28493	26.82	7.24	6.38	8.20	4.80	3.75	6.14	8.82	7.59	10.24
60–64	15699	14.78	9.14	8.05	10.39	5.22	4.06	6.72	11.35	9.75	13.21
Sex											
Women	32876	30.95	1.00			1.00			1.00		
Men	73353	69.05	1.48	1.41	1.56	0.73	0.66	0.81	1.78	1.69	1.89
Married											
No	22280	20.97	1.00			1.00			1.00		
Yes	83949	79.03	1.13	1.06	1.20	0.94	0.83	1.06	1.21	1.12	1.30
Dependent children											
No	36939	34.77	1.00			1.00			1.00		
Yes	69290	65.23	0.91	0.87	0.96	0.92	0.83	1.03	0.91	0.86	0.96
Annual salary											
< 40 K	19337	18.2	1.00			1.00			1.00		
40 K- < 70 K	28115	26.47	1.25	1.17	1.34	1.23	1.07	1.41	1.23	1.14	1.33
70 K- < 100 K	23763	22.37	1.31	1.22	1.41	1.25	1.07	1.45	1.28	1.19	1.39
≥ 100 K	27456	25.85	1.29	1.20	1.38	1.51	1.31	1.75	1.21	1.12	1.30
Missing	7558	7.11	1.01	0.92	1.10	1.18	0.98	1.43	0.99	0.90	1.10
Year											
2016	20655	19.44	1.00			1.00			1.00		
2017	21360	20.11	1.07	1.00	1.14	1.00	0.88	1.14	1.08	1.01	1.16
2018	21835	20.55	1.14	1.07	1.21	0.97	0.86	1.11	1.18	1.10	1.26
2019	21352	20.10	1.14	1.07	1.21	0.94	0.83	1.08	1.20	1.12	1.28
2020	21027	19.79	1.12	1.05	1.19	0.91	0.80	1.04	1.16	1.09	1.24

^†^Adjusted for age, sex, marital status, dependent children, salary, and year

### Statistical techniques

Numbers and percentages were used to describe the variables. Rates of mental disorders, sleep disorders, and medication use were calculated. Rates of sleep disorders were also calculated, conditioned on having one of the selected mental disorders. Odds ratios were used to measure associations between sleep disorders and mental disorders (with and without comorbid mental disorders) and the association between sleep disorders and selected types of mental health medication. We used the method of maximum likelihood to estimate parameters in a logistic regression model. The model adjusted for potential confounders: age, sex, marital status, dependent children status, salary, year, and in some cases mental disorders. From the estimated model, we derived adjusted odds ratios and corresponding 95% confidence intervals. Two-sided tests of significance were used. If the bound of the 95% confidence interval for the odds ratio estimate does not contain 1, it is statistically significant at the 0.05 level. Statistical analyses were derived from Statistical Analysis System (SAS) software, version 9.4 (SAS Institute Inc., Cary, NC, USA, 2012).

## Results

Among contract holders, 11.7% filed one or more claims for a sleep disorder. The two most common types of sleep disorders for which healthcare claims were submitted are insomnia (2.2%) and sleep apnea (9.7%). The odds of insomnia significantly increase with age (*p* < 0.0001), are higher in women (*p* < 0.0001) and in contract holders with incomes of at least $40,000 per year (*p* < 0.0001) (Table 1). There are no significant interactions among these variables in their associations with insomnia. The odds of sleep apnea significantly increase with age (more so than with insomnia) (*p* < 0.0001), are higher in men (*p* < 0.0001), married (*p* < 0.0001), in contract holders without dependent children (*p* = 0.0010), and in contract holders making at least $40,000 per year (*p* < 0.0001). Significant interactions exist involving sex and age (*p* = 0.0062) and sex and marital status (*p* < 0.0001). Specifically, the increase in sleep apnea with age is greater for women than men, and while there is no association between marital status and sleep apnea in women, there is a positive association in men (data not shown).

Rates of filing one or more claims for selected mental disorders range from 0.09% for schizophrenia to 8.4% for anxiety (Table 2). Compared with the overall rates of insomnia and sleep apnea mentioned above, the rates of these sleeping disorders is much greater for those with any one of the mental disorders shown. Insomnia rates are greatest in those with bipolar disorder or schizophrenia. Sleep apnea rates are greatest for those with bipolar disorder or depression. Sleep apnea rates are over two times greater than the insomnia rates for each of the mental disorders, except for schizophrenia where it is only slightly higher for those patients with sleep apnea.

**Table 2:** Mental disorders correspond with comorbid sleep disorders

	No.	%	Comorbid sleep disorder		Comorbid insomnia		Comorbid sleep apnea	
			No.	%	No.	%	No.	%
Stress	2114	1.99	416	19.68	126	5.96	315	14.90
Anxiety	8902	8.38	2128	23.90	706	7.93	1531	17.20
Depression	8248	7.76	2184	26.48	552	6.69	1742	21.12
ADHD	1975	1.86	460	23.29	121	6.13	372	18.84
Bipolar Disorder	646	0.61	218	33.75	75	11.61	163	25.23
OCD	370	0.35	74	20.00	25	6.76	57	15.41
Schizophrenia	92	0.09	26	28.26	14	15.22	15	16.30

ADHD, attention-deficit hyperactivity disorder; OCD, obsessive–compulsive disorder

The level of comorbid mental disorders for stress is 51.7%, for anxiety is 48.2%, for depression is 51.4%, for ADHD is 47.6%, for bipolar disorder is 68.4%, for OCD is 71.6%, and for schizophrenia is 87.0%. The odds of having a sleep disorder are significantly greater (*p* < 0.05) in those with a mental disorder, especially if the index mental disorder is associated with comorbid mental disorders (Table 3).

**Table 3:** Odds of sleep disorders according to selected mental disorders (with and without comorbid mental disorders)

	No.	%	Any sleep disorder			Insomnia			Sleep apnea		
			Odds ratio^†^	95% LCL^†^	95% UCL^†^	Odds ratio^†^	95% LCL^†^	95% UCL^†^	Odds ratio^†^	95% LCL^†^	95% UCL^†^
No mental disorder	90083	97.71	1.00			1.00			1.00		
Stress only	1021	1.11	1.63	1.35	1.97	2.55	1.81	3.60	1.44	1.16	1.78
Stress +	1093	1.19	4.19	3.63	4.84	6.68	5.33	8.38	3.50	2.98	4.10
No mental disorder	90,083	91.01	1.00			1.00			1.00		
Anxiety only	4613	4.66	2.73	2.52	2.95	5.69	5.02	6.46	1.99	1.82	2.18
Anxiety +	4289	4.33	4.69	4.35	5.06	7.43	6.57	8.41	3.86	3.55	4.19
No mental disorder	90,083	91.61	1.00			1.00			1.00		
Depression only	4012	4.08	3.32	3.07	3.59	3.47	2.97	4.05	3.09	2.84	3.36
Depression +	4236	4.31	4.76	4.41	5.13	6.94	6.12	7.87	4.03	3.72	4.37
No mental disorder	90,083	97.85	1.00			1.00			1.00		
ADHD only	1034	1.12	2.35	1.99	2.77	4.41	3.32	5.87	2.07	1.72	2.48
ADHD +	941	1.02	4.72	4.06	5.49	6.28	4.86	8.12	4.22	3.60	4.96
No mental disorder	90,083	99.29	1.00			1.00			1.00		
Bipolar only	204	0.22	3.88	2.81	5.36	7.01	4.22	11.64	3.02	2.13	4.30
Bipolar +	442	0.49	6.27	5.09	7.71	10.75	8.06	14.33	4.71	3.77	5.89
No mental disorder	90,083	99.59	1.00			1.00			1.00		
OCD only	105	0.12	2.34	1.37	4.02	8.46	4.21	17.00	2.01	1.10	3.67
OCD +	265	0.29	3.89	2.86	5.30	6.31	3.77	10.58	3.31	2.36	4.65
No mental disorder	90,083	99.9	1.00			1.00			1.00		
Schizophrenia only	12	0.01	1.61	0.34	7.55	12.12	2.61	56.22	–-		
Schizophrenia +	80	0.09	4.87	2.95	8.03	13.71	7.30	25.75	2.99	1.67	5.37

^†^Adjusted for age, sex, marital status, dependent children, salary, and year

-Insufficient numbers to compute

**Fig. 1:**
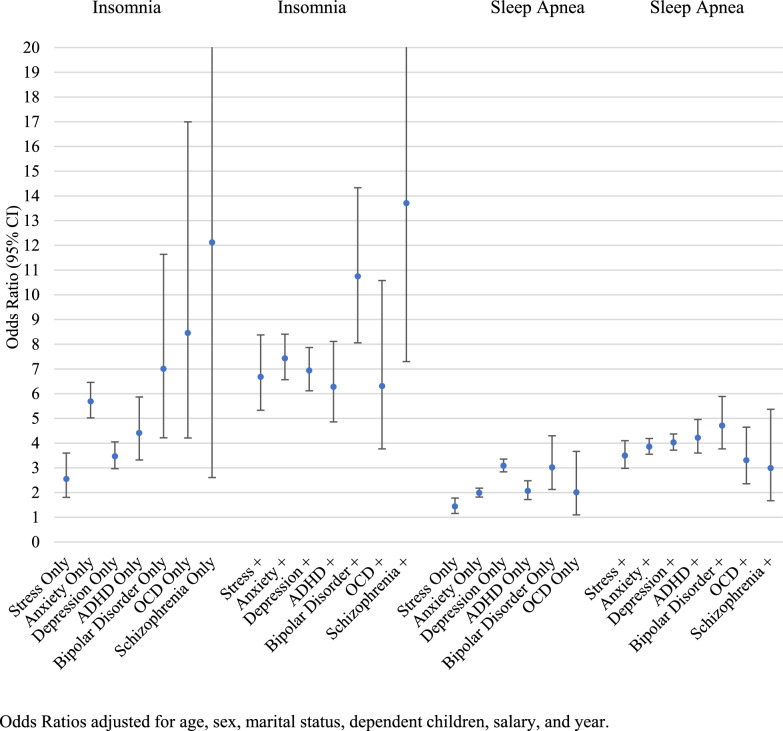
Odds of insomnia and sleep apnea according to selected mental disorders (with and without comorbid mental disorders)

The results shown in the table are also presented graphically (Fig. 1). This graph emphasizes that the association between mental disorders and insomnia or mental disorders and sleep apnea is strong, especially when the mental disorders have comorbid mental illness. The graph also shows that mental disorders are more strongly associated with insomnia than with sleep apnea.

The rate of medication use among contract holders ranges from 0.6% for CNS stimulants to 16.3% for psychostimulants (Table 4). Each mental disorder, except ADHD, is treated primarily using psychostimulants, ataractics, and anticonvulsants. ADHD patients are generally treated with amphetamines. This can also be assessed differently by assessing which mental disorders are most likely treated using each type of medication. CNS stimulants and amphetamines are most commonly used to treat ADHD; psychostimulants are commonly used to treat each type of mental disorder; sedatives (non-barbiturate) are most commonly used to treat bipolar disorder, anxiety, depression, and schizophrenia; and ataractics and anticonvulsants are most commonly used to treat bipolar disorder and schizophrenia.

**Table 4:** Medication use for selected mental disorders, 2016–2020

Medication type	CNS stimulants	Psychostimulants (antidepressants)	Amphetamines	Sedatives (non-barbiturate)	Ataractics	Anticonvulsants
No.	589	17,288	2,472	4,522	5,683	6,931
%	0.55 %	16.27 %	2.33 %	4.26 %	5.35 %	6.52 %
Stress	1.32	41.72	6.39	8.75	18.50	17.74
Anxiety	2.11	65.19	7.85	12.29	27.23	22.06
Depression	2.56	76.43	9.69	12.18	20.49	22.22
ADHD	14.53	49.47	77.97	9.52	14.73	16.56
Bipolar	3.41	76.47	16.1	21.36	57.28	73.07
OCD	2.16	78.38	11.62	7.03	26.49	20.81
Schizophrenia	3.26	70.65	6.52	13.04	82.61	39.13

The odds of a sleep disorder (yes vs. no) for selected psychotropic drugs appear in Table 5. The odds of insomnia and of sleep apnea are significantly positively associated with each of the medication classifications (*p* < 0.05), after adjusting for age, sex, marital status, dependent children, salary, and year. Insomnia tends to be more strongly associated with psychotropic drug use, particularly sedatives (non-barbiturate). After further adjustment for the mental disorders, the odds ratios decrease, but remain significantly positively associated with insomnia and sleep apnea (*p* < 0.05), except for CNS stimulants. For insomnia and sleep apnea, the greatest percent decrease in odds ratios involves CNS stimulants and amphetamines.

**Table 5:** Odds of a sleep disorder according to mental health medication use, 2016–2020

	Any sleep disorder			Insomnia			Sleep apnea		
	Odds ratio^†^	95% LCL^†^	95% UCL^†^	Odds ratio^†^	95% LCL^†^	95% UCL^†^	Odds ratio^†^	95% LCL^†^	95% UCL^†^
CNS stimulants	3.30	2.36	4.61	3.84	2.07	7.10	2.40	1.96	2.95
Psychostimulants (antidepressants)	4.06	3.84	4.30	6.53	5.83	7.32	3.53	3.38	3.70
Amphetamines	3.09	2.52	3.79	3.54	2.39	5.24	2.78	2.51	3.09
Sedatives (non-barbiturate)	5.40	4.98	5.84	34.64	30.75	39.03	2.38	2.21	2.56
Ataractics	3.01	2.75	3.29	5.06	4.34	5.91	2.57	2.40	2.76
Anticonvulsants	3.12	2.89	3.36	4.61	4.01	5.29	2.94	2.76	3.12

	Odds Ratio^‡^	95% LCL^‡^	95% UCL^‡^	Odds Ratio^‡^	95% LCL^‡^	95% UCL^‡^	Odds Ratio‡	95% LCL‡	95% UCL‡
CNS stimulants	2.73	1.93	3.87	0.98	0.67	1.44	1.19	0.95	1.49
Psychostimulants (antidepressants)	3.26	3.10	3.43	4.50	4.06	4.98	2.82	2.67	2.98
Amphetamines	1.95	1.69	2.24	1.45	1.14	1.86	1.74	1.50	2.02
Sedatives (non-barbiturate)	4.51	4.22	4.82	21.49	19.53	23.64	1.86	1.73	2.01
Ataractics	2.02	1.88	2.17	2.73	2.43	3.07	1.70	1.58	1.84
Anticonvulsants	2.39	2.25	2.54	2.49	2.23	2.78	2.20	2.06	2.35

^†^Adjusted for age, sex, marital status, dependent children, salary, and year

^‡^Adjusted for age, sex, marital status, dependent children, salary, year, and the mental disorders

**Fig. 2:**
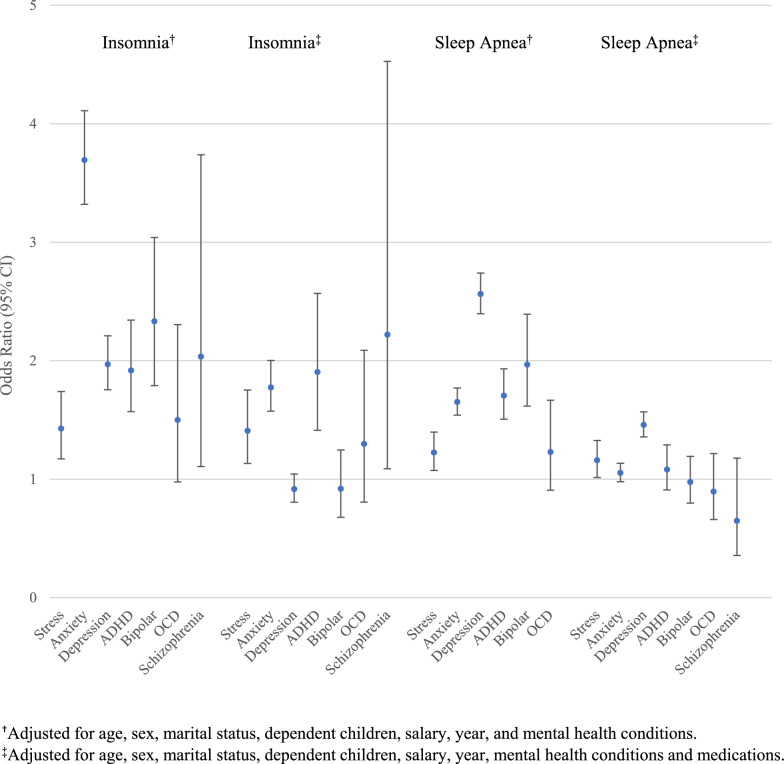
Odds of insomnia and sleep apnea according to selected mental disorders

The odds ratios show the strength of the association between each of the mental disorders and insomnia, as well as between the mental disorders and sleep apnea (Fig. 2). The odds ratios are adjusted for either demographics and mental disorders or demographics, mental disorders, and psychotropic drugs (see Table 4 for a list of medications). Each of the mental disorders, except for OCD, is significantly associated with both insomnia and sleep apnea, after adjusting for demographics and other mental disorders. With further adjustment for mental health medication, the odds ratios tend to decrease. For example, the odds of insomnia is 3.7 times greater in those with anxiety compared to those without anxiety prior to the additional adjustment for psychotropic drugs, but 1.8 times greater after the additional adjustment for medication. The odds of sleep apnea are 1.7 times greater in those with anxiety compared with those without anxiety prior to the additional adjustment for psychotropic drugs, but 1.1 times greater after the additional adjustment for medication.

## Discussion

This study evaluated whether sleep disorders positively associate with selected index mental disorders; if a positive association between a sleep disorder and mental disorder differs according to whether there was one versus multiple mental disorders; and whether psychotropic drug use increases the prevalence of sleep disorders, after adjusting for selected variables.

Rates of insomnia and sleep apnea increased with age, more so for sleep apnea. Higher rates of these disorders with increasing age are consistent with previous studies [18, 19, 41]. Sex differences for sleep disorders vary, depending on the specific sleep disorder. Consistent with previous research, insomnia is more common in women, and sleep apnea is more common in men [20–22, 41]. Higher sleep apnea rates among married versus single adults may be because having a sleeping partner increases the chance of detecting the disorder.

Support for our first hypothesis exists because each mental disorder is positively associated with the sleep disorders. This result is consistent with previous studies [9–15]. Insomnia had the strongest association with bipolar disorder and schizophrenia. Insomnia may be more common in these mental disorders because of psychotic symptoms causing intense fear and anxiety. Sleep apnea had the strongest association with depression and bipolar disorder. Sleep apnea may be more strongly associated with bipolar and depression because of its bi-directional relationship [42].

We next considered whether these associations were further increased for the index mental disorder if it was associated with comorbid mental disorders. Each mental disorder had a high percentage of comorbid mental illness, as consistent with previous studies [43–46]. The mental disorders that are generally considered to have a greater impact on daily life, such as schizophrenia, OCD, and bipolar disorder, had higher levels of comorbid mental disorders, as also consistent with previous studies [24–28]. Supporting our second hypothesis, individuals with both index mental disorders associated and comorbid mental disorders tended to have even greater levels of insomnia and sleep disorders, as consistent with previous studies [47, 48].

Finally, in support of our third hypothesis, each psychotropic drug was positively associated with insomnia and sleep apnea, even after adjusting for age, sex, and comorbid mental disorders. This is consistent with previous research indicating a positive association directly between psychotropic drugs and sleep disorders [29–36]. Psychotropic drugs are generally more strongly associated with insomnia than sleep apnea. It is not clear from our data the extent to which the drug resulted in the sleeping disorder or the sleeping disorder led to the prescribed drug.

Psychostimulants are the most commonly used medication for treating mental disorders, except for ADHD, where amphetamine preparations are most common. Both findings are consistent with the literature [49, 50]. Anticonvulsants are almost as commonly used in bipolar patients as psychostimulants. Previous studies found that psychostimulants are most commonly prescribed for bipolar disorder, ranging from 70%-72% [41, 51–53]. Ataractics, also known as antipsychotics, are more commonly prescribed than psychostimulants for schizophrenia. This is consistent with current medical practice [16, 54].

Each of the mental disorders, except OCD, positively associated with insomnia, after adjusting for demographics and other mental disorders. Further adjustment for psychotropic drugs significantly decreases the association between the mental disorders anxiety, depression, and bipolar disorder with insomnia. Depression and bipolar disorder are no longer associated with insomnia in the fully adjusted model. Hence, insomnia among people with anxiety, depression, or bipolar disorder appear to be higher because of the psychotropic drugs used to treat these mental health problems. Except for CNS stimulants, all the medications are implicated, primarily sedatives (non-barbiturate) followed by psychostimulants. The relatively strong positive association of insomnia with sedatives (non-barbiturate) is a unique finding. However, the weak association with amphetamine preparations has been shown previously [31]. There is little change in the association between stress, ADHD, OCD, and schizophrenia with insomnia, indicating that medication use has little influence on the increased risk of insomnia for these mental disorders. The positive association that remains between stress, ADHD, and schizophrenia with insomnia indicates that the increased risk of insomnia is almost entirely attributed to these mental disorders and not their associated medications.

Each of the mental disorders is positively associated with sleep apnea, except OCD and schizophrenia (which have insufficient numbers to assess), after adjusting for demographics and other mental disorders. Further adjustment for psychotropic drugs significantly decreased the association between the mental disorders anxiety, depression, ADHD, and bipolar disorder with sleep apnea, to the point that the association becomes insignificant for anxiety and ADHD. Hence, psychotropic drugs explain some of the positive association for depression and bipolar disorder and all the association for anxiety and ADHD. Except for CNS stimulants, all the medications are implicated in the positive association, especially psychostimulants and then anticonvulsants. The positive association between stress and sleep apnea is not affected by psychotropic drugs.

### Limitation

A mental health disorder or sleep disorder diagnosis would not lead to a loss of insurance, making selective under-reporting of claims unlikely. One or more claims were used to identify the presence of each of the mental disorders. However, less serious cases, which did not lead to medical attention and resulting claims, are not represented. Hence, measures of association between mental disorders and sleep disorders may be biased downward. Finally, the current study was limited to identifying associations and not causal relationships. The causal direction between mental disorders and sleep disorders was two-way, but the extent of this bi-directional relationship could not be determined in our data. However, beyond stress, anxiety, and depression, it is likely that the mental disorders led to the sleep disorders.

## Conclusion

Mental disorders positively correlate with insomnia and sleep apnea, more so with insomnia. A high level of comorbid mental illness is associated with the index mental disorders. The positive associations between mental disorders and insomnia and sleep apnea are greater when comorbid mental illness exists. Bipolar disorder and schizophrenia (alone or with comorbid mental disorders) are most strongly associated with insomnia, and bipolar disorder and depression are most strongly associated with sleep disorders.

Psychotropic drugs other than CNS stimulants, primarily sedatives (non-barbiturate) and psychostimulants, account for some of the positive association between anxiety and insomnia and all of the association between depression and insomnia and bipolar disorder and insomnia. Medication use has little influence on the increased risk of insomnia due to stress, ADHD, OCD, and schizophrenia. Psychotropic drugs account for some of the positive association between depression and sleep apnea and bipolar disorder and sleep apnea and all the association for anxiety and ADHD with sleep apnea. All the medications except CNS stimulants account for some of the positive associations with sleep apnea, especially psychostimulants, and then anticonvulsants. The positive association between stress and sleep apnea is not affected by psychotropic drugs.

## Data Availability

Data used to generate the results in this study are available to any scientist wishing to use them for non-commercial purposes upon request to the lead author.

## Abbreviations

ADHD: Attention deficit hyperactivity disorder
ASD: Autism spectrum disorder
DMBA: Deseret Mutual Benefit Administrators
HIPAA: Health Insurance Portability and Accountability Act
ICD-10-CM: International Classification of Diseases: Tenth Revision: Clinical Modification
OCD: Obsessive–compulsive disorder
REM: Rapid eye movement
SAS: Statistical analysis system

## References

[1] American Psychiatric Association. What are sleep disorders? 2020. https://www.psychiatry.org/patients-families/sleep-disorders/what-are-sleep-disorders. Accessed 10 Jun 2022.

[2] Medic G, Wille M, Hemels ME (2017). Short- and long-term health consequences of sleep disruption. Nat Sci Sleep.

[3] Lim J, Dinges DF (2010). A meta-analysis of the impact of short-term sleep deprivation on cognitive variables. Psychol bull.

[4] Besedovsky L, Lange T, Born J (2012). Sleep and immune function. Pflugers Arch.

[5] Kono M, Tatsumi K, Saibara T, Nakamura A, Tanabe N, Takiguchi Y (2007). Obstructive sleep apnea syndrome is associated with some components of metabolic syndrome. Chest.

[6] Khandelwal D, Dutta D, Chittawar S, Kalra S (2017). Sleep disorders in type 2 diabetes. Indian J Endocrinol Metab.

[7] Covassin N, Singh P (2016). Sleep duration and cardiovascular disease risk: epidemiologic and experimental evidence. Sleep Med Clin.

[8] Taylor DJ, Mallory LJ, Lichstein KL, Durrence HH, Riedel BW, Bush AJ (2007). Comorbidity of chronic insomnia with medical problems. Sleep.

[9] Almojali AI, Almakli SA, Alothman AS, Masuadi EM, Alaqeel MK (2017). The prevalence and association of stress with sleep quality among medical students. J Epidemiol Glob Health.

[10] Baglioni C, Nanovska S, Regen W, Spiegelhalder K, Feige B, Nissen C (2016). Sleep and mental disorders: a meta-analysis of polysomnographic research. Psychol Bull.

[11] Wong JL, Martinez F, Aguila AP, Pal A, Aysola RS, Henderson LA (2021). Stress in obstructive sleep apnea. Sci Rep.

[12] Becker SP, Cusick CN, Sidol CA, Epstein JN, Tamm L (2018). The impact of comorbid mental health symptoms and sex on sleep functioning in children with ADHD. Eur Child Adolesc Psychiatry.

[13] Buysse DJ, Angst J, Gamma A, Ajdacic V, Eich D, Rössler W (2008). Prevalence, course, and comorbidity of insomnia and depression in young adults. Sleep.

[14] Krystal AD (2012). Psychiatric disorders and sleep. Neurol Clin.

[15] Kalmbach DA, Anderson JR, Drake CL (2018). The impact of stress on sleep: Pathogenic sleep reactivity as a vulnerability to insomnia and circadian disorders. J Sleep Res.

[16] Laskemoen JF, Simonsen C, Büchmann C, Barrett EA, Bjella T, Lagerberg TV (2019). Sleep disturbances in schizophrenia spectrum and bipolar disorders—a transdiagnostic perspective. Compr Psychiatry.

[17] Freeman D, Taylor KM, Molodynski A, Waite F (2019). Treatable clinical intervention targets for patients with schizophrenia. Schizophr Res.

[18] Ferrarelli F (2021). Sleep abnormalities in schizophrenia: state of the art and next steps. Am J Psychiatry.

[19] Hvolby A (2015). Associations of sleep disturbance with ADHD: implications for treatment. Atten Defic Hyperact Disord.

[20] Cox RC, Olatunji BO (2022). Delayed circadian rhythms and insomnia symptoms in obsessive-compulsive disorder. J Affect Disord.

[21] Fang H, Tu S, Sheng J, Shao A (2019). Depression in sleep disturbance: a review on a bidirectional relationship, mechanisms and treatment. J Cell Mol Med.

[22] Staner L (2003). Sleep and anxiety disorders. Dialogues Clin Neurosci.

[23] van den Akker M, Buntinx F, Knottnerus JA (1996). Comorbidity or multimorbidity: what's in a name? A review of literature. Eur J Gen Pract.

[24] Andrews G, Slade T, Issakidis C (2002). Deconstructing current comorbidity: data from the Australian national survey of mental health and well-being. Br J Psychiatry.

[25] Bland RC (1992). Psychiatric disorders in America: the epidemiologic catchment area study. J Psychiatry Neurosci.

[26] Bijl RV, Ravelli A, van Zessen G (1998). Prevalence of psychiatric disorder in the general population: results of the Netherlands mental health survey and incidence study (NEMESIS). Soc Psychiatry Psychiatr Epidemiol.

[27] Plana-Ripoll O, Pedersen CB, Holtz Y, Benros ME, Dalsgaard S, de Jonge P (2019). Exploring comorbidity within mental disorders among a Danish national population. JAMA Psychiat.

[28] Kessler RC, Chiu WT, Demler O, Merikangas KR, Walters EE (2005). Prevalence, severity, and comorbidity of 12-month DSM-IV disorders in the national comorbidity survey replication. Arch Gen Psychiatry.

[29] Gahr M, Connemann BJ, Zeiss R, Fröhlich A (2018). Sleep disorders and impaired sleep as adverse drug reactions of psychotropic drugs: an evaluation of data of summaries of product characteristics. Fortschr Neurol Psychiatr.

[30] Wichniak A, Wierzbicka A, Walęcka M, Jernajczyk W (2017). Effects of antidepressants on sleep. Curr Psychiatry Rep.

[31] Pagel JF, Parnes BL (2001). Medications for the treatment of sleep disorders: an overview. Prim Care Companion J Clin Psychiatry.

[32] DeMartinis NA, Winokur A (2007). Effects of psychiatric medications on sleep and sleep disorders. CNS Neurol Disord Drug Targets.

[33] Rush AJ, Armitage R, Gillin JC, Yonkers KA, Winokur A (1998). Comparative effects of nefazodone and fluoxetine on sleep in outpatients with major depressive disorder. Biol Psychiatry.

[34] Chen L, Bell JS, Visvanathan R, Hilmer SN, Emery T, Robson L (2016). The association between benzodiazepine use and sleep quality in residential aged care facilities: a cross-sectional study. BMC Geriatr.

[35] Doghramji K, Jangro WC (2016). Adverse effects of psychotropic medications on sleep. Psychiatr Clin North Am.

[36] Van Gastel A (2018). Drug-induced insomnia and excessive sleepiness. Sleep Med Clin.

[37] Suni E. Sleep Statistics. Sleep Foundation. https://www.sleepfoundation.org/how-sleep-works/sleep-facts-statistics. Accessed 14 Oct 2022.

[38] Torrens Darder I, Argüelles-Vázquez R, Lorente-Montalvo P, Torrens-Darder MDM, Esteva M (2021). Primary care is the frontline for help-seeking insomnia patients. Eur J Gen Pract.

[39] Al-Shawwa B, Glynn E, Hoffman MA, Ehsan Z, Ingram DG (2021). Outpatient health care utilization for sleep disorders in the Cerner Health Facts database. J Clin Sleep Med.

[40] World Health Organization. ICD-10: International statistical classification of diseases and related health problems: tenth revision, 2nd ed; 2004. https://apps.who.int/iris/handle/10665/42980. Accessed 10 Jun 2022.

[41] Lin CM, Davidson TM, Ancoli-Israel S (2008). Gender differences in obstructive sleep apnea and treatment implications. Sleep Med Rev.

[42] Jehan S, Auguste E, Pandi-Perumal SR, Kalinowski J, Myers AK, Zizi F, Rajanna MG, Jean-Louis G, McFarlane SI (2017). Depression, obstructive sleep apnea and psychosocial health. Sleep Med Disord.

[43] Katzman MA, Bilkey TS, Chokka PR, Fallu A, Klassen LJ (2017). Adult ADHD and comorbid disorders: clinical implications of a dimensional approach. BMC Psychiatry.

[44] Sharma E, Sharma LP, Balachander S, Lin B, Manohar H, Khanna P (2021). Comorbidities in obsessive-compulsive disorder across the lifespan: a systematic review and meta-analysis. Front Psychiatry.

[45] Buckley PF, Miller BJ, Lehrer DS, Castle DJ (2009). Psychiatric comorbidities and schizophrenia. Schizophr Bull.

[46] Hirschfeld RM (2001). The comorbidity of major depression and anxiety disorders: recognition and management in primary care. Prim Care Companion J Clin Psychiatry.

[47] Roth T (2007). Insomnia: definition, prevalence, etiology, and consequences. J Clin Sleep Med.

[48] Blank M, Zhang J, Lamers F, Taylor AD, Hickie IB, Merikangas KR (2015). Health correlates of insomnia symptoms and comorbid mental disorders in a nationally representative sample of US adolescents. Sleep.

[49] Saha K, Sugar B, Torous J, Abrahao B, Kıcıman E, De Choudhury M (2019). A social media study on the effects of psychiatric medication use. Proc Int AAAI Conf Weblogs Soc Media.

[50] Connolly JJ, Glessner JT, Elia J, Hakonarson H (2015). ADHD & pharmacotherapy: past, present and future: a review of the changing landscape of drug therapy for attention deficit hyperactivity disorder. Ther Innov Regul Sci.

[51] Zhang B, Wing YK (2006). Sex differences in insomnia: a meta-analysis. Sleep.

[52] Bellivier F, Delavest M, Coulomb S, Figueira ML, Langosch JM, Souery D (2014). Prise en charge thérapeutique des patients présentant un trouble bipolaire en France et en Europe : étude multinationale longitudinale WAVE-bd [Therapeutic management of bipolar disorder in France and Europe: A multinational longitudinal study (WAVE-bd)]. Encephale.

[53] Baldessarini R, Henk H, Sklar A, Chang J, Leahy L (2008). Psychotropic medications for patients with bipolar disorder in the United States: Polytherapy and adherence. Psychiatr Serv.

[54] Mayo Clinic. Schizophrenia. Diseases & Conditions. https://www.mayoclinic.org/diseases-conditions/schizophrenia/diagnosis-treatment/drc-20354449. Accessed 6 Jun 2022.

